# Two Cases of Bi-Lateral Lithotomy

**Published:** 1876-04

**Authors:** John S. Coleman

**Affiliations:** Augusta, Ga.


### TWO CASES OF BI-LATERAL LITHOTOMY.

By JOHN S. COLEMAN, M.D., Augusta, Ga.

Case I.—Adam, colored, æt. fifty-two years, was sent from Aiken, S. C., to Dr. Edward Geddings, of this city, to be treated for stricture. After fully dilating the canal, the Doctor found that he was also the subject of vesical calculi. Not caring to operate himself, Dr. Geddings kindly transferred the case to my care.

June 12, 1873. The patient still suffers from spasmodic stricture, which materially interferes with the introduction of instruments. He is unable to lie upon his back, and only for a short time in bed; is constantly on his knees, leaning forward upon some object; constant and painful micturition.

June 17, 1873. Assisted by Drs. Campbell, Geddings, Eve, and Foster, I made the bi-lateral section with the double lithotome. Upon introducing my finger into the bladder, I found that there were two stones, one very large and the other small. The latter was readily removed, although partially crushed by the forceps. With the large stone I found it necessary to extend the incision, with the probe-pointed bistoury, on both sides of the prostate, and then could only extract it after the expenditure of considerable force. The compression of the forceps was such as to crumble into sand about one-eighth of its surface. Measurement of large stone, 1 7-8 by 1 1-16 inches; weight 783 grains. Measurement of small stone, 9-16 by 1 1-16 inches; weight 27 grains; total 810 grains. This exclusive of the crumbled particles, as they were not preserved. Analysed by Prof. G. W. Rains, they were found to be composed of the triple phosphates.

July 2. Wound closed.

July 9. Patient discharged, well.

Case II.—H. H., white, æt. forty-five, was referred to me by a professional friend, for the treatment of a stricture. This I fully dilated by the continuous and multiple wedge methods. Some days afterwards, on passing a steel sound, I discovered the existence of two small stones; one being in the prostatic portion of the urethra and the other in the bladder. On three different occasions I thought that I had succeeded in pushing the one in the urethra back into the bladder; for I could then distinctly feel the two in that viscus, and at the same time failed to feel, as before, the grating of that in the urethra against the sound.

At 11 a.m., on the 12th of February, 1876, with the assistance of several medical friends, and in the presence of the class of the medical department of the University of Georgia,

I performed the bi-lateral operation. On introducing the forceps, I readily grasped together and removed the two stones in the bladder. I now carefully explored the bladder with the finger, and found that there were none remaining in it, but that there were two other stones in a large pouch in the prostatic urethra. These I could not grasp with the forceps, as they were above the line of my incision. I therefore, after some effort, removed them with the finger. The stones are of irregular shape, faceted, and of nearly uniform size; the weights being respectively 16, 13, 13, and 12½ grains. Composition phosphate of lime.

14th. H. has been considerably troubled with nausea and vomiting. All the urine now passes through the urethra, and is tinged with blood.

17th. The urine again passes through the wound. As the bowels have not been moved since the operation, the patient was ordered to take one ounce of castor oil.

March 9th. On the fourteenth day the urine ceased to pass through the wound. H. has suffered from a severe attack of malarial fever. Is now convalescent.

11th. Wound closed. Patient discharged from hospital.

It is worthy of remark that both patients were suffering from stricture, and that in one (H.) the existence of stone was not even suspected.

